# Genomic landscape of pleural and peritoneal mesothelioma tumours

**DOI:** 10.1038/s41416-022-01979-0

**Published:** 2022-09-22

**Authors:** Stefanie Hiltbrunner, Zoe Fleischmann, Ethan S. Sokol, Martin Zoche, Emanuela Felley-Bosco, Alessandra Curioni-Fontecedro

**Affiliations:** 1 Department of Medical Oncology and Hematology, University Hospital Zurich, Zurich, Switzerland; 2 University of Zurich, Zurich, Switzerland; 3 Foundation Medicine, Cambridge, MA USA; 4 Pathology Department, University Hospital Zurich, 8091 Zurich, Switzerland; 5 Laboratory of Molecular Oncology, Department of Thoracic Surgery, University Hospital Zurich, Zurich, Switzerland

**Keywords:** Mesothelioma, Cancer genetics

## Abstract

**Background:**

Malignant pleural and peritoneal mesotheliomas are rare malignancies with unacceptable poor prognoses and limited treatment options. The genomic landscape is mainly characterised by the loss of tumour suppressor genes and mutations in DNA repair genes. Currently, data from next-generation sequencing (NGS) of mesothelioma tumours is restricted to a limited number of cases; moreover, data comparing molecular features of mesothelioma from the pleural and peritoneal origin with NGS are lacking.

**Methods:**

We analysed 1113 pleural mesothelioma and 355 peritoneal mesothelioma samples. All tumours were sequenced with the FoundationOne® or FoundationOne®CDx assay for detection of substitutions, insertion–deletions, copy-number alterations and selected rearrangements in at least 324 cancer genes.

**Results:**

This analysis revealed alterations in 19 genes with an overall prevalence of at least 2%. Alterations in *BAP1, CDKN2A, CDKN2B, NF2, MTAP, TP53* and *SETD2* occurred with a prevalence of at least 10%. Peritoneal, compared to pleural mesothelioma, was characterised by a lower prevalence of alterations in *CDKN2A, CDKN2B* and *MTAP*. Moreover, we could define four distinct subgroups according to alterations in *BAP1* and *CDKN2A/B*. Alterations in Hedgehog pathway-related genes (*PTCH1/2* and *SUFU*) and Hippo pathway-related gene (*NF2)* as well as *KRAS, EGFR, PDGFRA/B, ERBB2* and *FGFR3* were detected in both cohorts.

**Conclusion:**

Here, we report the molecular aberrations from the largest cohort of patients with mesothelioma. This analysis identified a proportion of patients with targetable alterations and suggests that molecular profiling can identify new treatment options for patients with mesothelioma.

## Introduction

Mesothelioma is a highly aggressive and lethal disease of the membrane lining of the serous cavities of the pleura, peritoneum, pericardium, and the tunica vaginalis testes. Malignant pleural mesothelioma is closely linked to asbestos and develops 30–40 years after initial exposure [1]. In comparison to pleural mesothelioma, peritoneal mesothelioma has only been modestly linked to asbestos [2]. Even though asbestos has been forbidden and strictly controlled in many countries, the global incidence of mesothelioma will rise in the coming years due to continued use in emerging economies and its long latency period.

Mesothelioma still has an unacceptably poor outcome, and there is great need for new therapeutic options. Since 2004, the first-line treatment consists of systemic combination therapy with pemetrexed and platinum-based chemotherapy or inclusion in clinical trials. Just recently, treatment with immune checkpoint inhibitors showed promising results and prolonged survival compared to standard chemotherapy treatment [3, 4]. The combination of ipilimumab and nivolumab was approved by the FDA in 2020 but is not yet a standard of care in most countries. In the later line setting, mesothelioma remains an orphan disease with very limited treatment options and other treatments such as multi-tyrosine kinase inhibitors sunitinib or sorafenib showed limited efficacy [5, 6], possibly related to the lack of patient stratification. Treatment allocation based on molecular features has been successful for advanced lung cancer, where targeted treatment options, even when applicable in 1–2% of cases, have improved patient outcomes [7]. This underlines the importance and potential of such alterations in mesothelioma, even if rare.

The genomic profile of mesothelioma reveals a low protein-coding mutation rate [8], with alterations of tumour suppressor genes and of epigenetic modifiers. The most commonly occurring alterations are deletions of the tumour suppressor genes *BAP1*, *NF2*, and *CDKN2A* [9]. Despite similarities of cellular features and common genetic alterations, pleural and peritoneal mesothelioma might be related to different risk factors; therefore, understanding the molecular features, which differentiate or associate these two diseases is crucial to give insights to the pathogenesis and treatment of these rare diseases. The aim of this study was to examine the prevalence of targetable alterations in pleural and peritoneal mesothelioma in a large cohort of cases; we report the mutational profile of 1468 mesothelioma patients, including the comparison of pleural and peritoneal mesothelioma profiled using NGS.

## Materials and methods

### Sequencing

Comprehensive genomic profiling (CGP) was performed with hybrid-capture-based next-generation sequencing on formalin-fixed and paraffin-embedded (FFPE) clinical specimen in a Clinical Laboratory Improvement Amendments-certified (CLIA), College of American Pathologists-accredited (CAP), New York State-approved laboratory (Foundation Medicine, Cambridge MA, more precise method description including percentage of tumour cell content, allele frequency, number of reads, copy-number detection as published in ref. [10]). CGP results included were from 1113 pleural mesothelioma and 355 peritoneal mesothelioma tumour tissue samples from patients in the United States sequenced through December 2020 as a part of routine clinical care. All tumours were sequenced with the FoundationOne^®^ or FoundationOne^®^CDx test for detection of substitutions, insertion–deletions, copy-number alterations and selected rearrangements in at least 324 cancer genes. Samples included in this study were sequenced to a median depth of 818× and mean depth of 782.2×. All known or likely pathogenic alterations were included in this study including heterozygous and homozygous somatic and germline alterations. Microsatellite instability was called on at least 95 loci [11], and tumour mutational burden (TMB) was calculated on at least 0.8 Mb as described in [12]. Tumours with high TMB were defined as those with at least 10 mutations per megabase. Approval for this study, including a waiver of informed consent and a HIPAA waiver of authorisation, was obtained from the Western Institutional Review Board (Protocol No. 20152817).

### Analysis

For the analysis of prevalence, the 95% confidence interval was determined using binomial error estimation. Co-occurrence and mutual exclusivity were analysed using Fisher’s exact test with multiple hypothesis test correction by Benjamini–Hochberg. Statistical significance was defined as *P* < 0.05. Gene prevalence in molecular subgroups was analysed by chi-square with multiple hypothesis correction by Benjamini–Hochberg.

## Results

### Cohort description

In total, 1113 pleural mesothelioma and 355 peritoneal mesothelioma samples from patients diagnosed through December 2020 were included in the analysis (total 994 male patients and 542 female patients) with 52.7% (*n* = 187) female, 47.3% male (*n* = 168) in the peritoneal group and 29.4% (*n* = 327) female, 70.6% (*n* = 786) male in the pleural group. Mesothelioma patients with other subtypes such as testis, pericardium and mesothelioma not otherwise specified were not included in this analysis.

### Landscape of genomic alterations in pleural and peritoneal mesothelioma

Mesothelioma is characterised by chromosomal abnormalities such as loss of chromosome regions and tumour suppressor genes [13], unlike other malignancies such as melanoma and lung cancer where the gain of function mutations in oncogenes are mainly present [14].

**Fig. 1:**
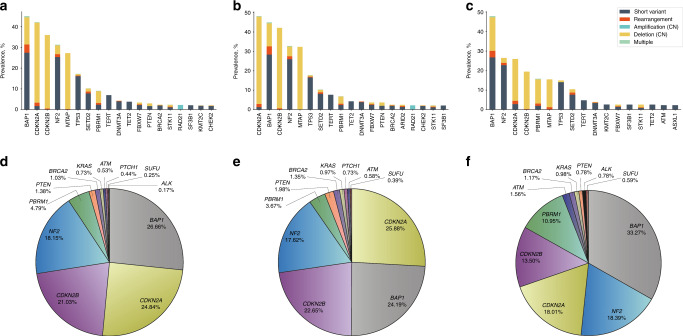
Genomic alterations in mesothelioma tumours. The prevalence of genomic alterations >2% in the entire cohort are represented: (**a**) prevalence of alterations in the entire cohort, (**b**) in pleural mesothelioma and (**c**) in peritoneal mesothelioma. The alterations include short variants (short nucleotide variants (SNV) and insertion–deletions (indels)), gene rearrangements and copy-number variations. Multiple alterations are defined as samples with more than one alteration in the same gene. Alterations of interest. Targetable alterations as well as mutations that define the four subgroups (Fig. 3) are shown in this pie plot as a percentage of all the alterations of interest (**d**) in the entire cohort, (**e**) in pleural mesothelioma and (**f**) in peritoneal mesothelioma.

We identified alterations with >2% prevalence in *BAP1* (45.1%)*, CDKN2A* (42.2%)*, CDKN2B* (36.0%)*, NF2* (31.3%)*, MTAP* (27.3%)*, TP53* (17.3%)*, SETD2* (10.2%)*, PBRM1* (9.1%)*, TERT* (7.0%)*, DNMT3A* (4.3%)*, TET2* (3.8%)*, FBXW7* (3.4%)*, PTEN* (3.0%)*, BRCA2* (2.3%)*, STK11* (2.3%)*, RAD21* (2.2%)*, SF3B1* (2.1%)*, KMT2C* (2.0%) and *CHEK2* (2.0%) in the total cohort (Fig. 1a). We could not detect any alterations in 55 (3.6%) of the cases (31 (2.8%) pleural mesothelioma and 21 (5.9%) peritoneal mesothelioma). *CDKN2A, CDKN2B* and *MTAP* alterations were primarily copy-number deletions (Fig. 1c), while the majority of other genes primarily consisted of short variant alterations. The five most frequently altered genes in pleural mesothelioma were *CDKN2A* (48.2%), *BAP1* (45.0%), *CDKN2B* (42.2%), *NF2* (32.8%) and *MTAP* (32.3%), in peritoneal mesothelioma *BAP1* (47.9%), *NF2* (26.5%), *CDKN2A* (25.9%), *CDKN2B* (19.5%), *PBRM1* (15.8%) (Fig. 1 and Supplementary Tables 1–3). In pleural mesothelioma tumours, we could detect significant differences between male and female patients in the prevalence of alterations in *CDKN2A/B*, *PBRM1* and *SF3B1*, while in peritoneal mesothelioma tumours there were no significant differences in the prevalence of the alterations (Supplementary Fig. 1).

**Fig. 2:**
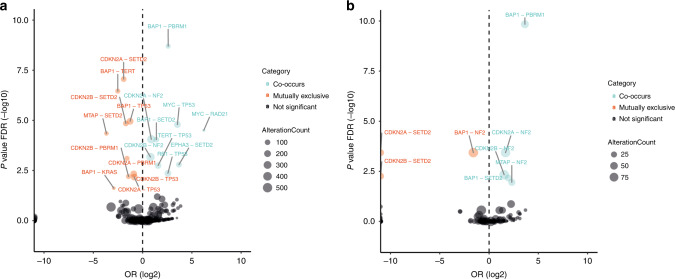
Correlation of molecular alterations. Co-occurring (blue) and mutually exclusive (red) alterations in **a** pleural and **b** peritoneal mesothelioma patients. The circle size marks the number of those alterations occurring.

In pleural and peritoneal mesothelioma, co-occurring alterations occurred on the same chromosome or in close proximity for *PBRM1–BAP1* (both 3p21) and *SETD2–BAP1* (both 3p21), which was observed in the TCGA cohort but not in the recent publication by Zauderer and colleagues [15]. However, more frequently, co-occurring alterations were not linked by their genetic loci like *NF2*-*CDKN2A* (22q12–9p21), *MYC–TP53* (8q24–17p13), *TERT–TP53* (5p15–17p13), *RB1–TP53* (13q14–17p13) (Fig. 2 and Supplementary Tables 4–6). This was also the case for mutually exclusive alterations such as *SETD2–CDKN2A* (3p21–9p21) and *TERT–BAP1* (5p15–3p21) (Fig. 2 and Supplementary Tables 7–9). *TP53* alterations co-occurred with *MYC, TERT* and *RB1* in pleural mesothelioma, indicating a subset of genomically unstable tumours, consistent with previous studies [9]. Interestingly, *CDKN2A/B* alterations did not co-occur with *TP53*, suggesting that permissive tumour growth conditions are gained with disruption of either of these two genes.

**Fig. 3:**
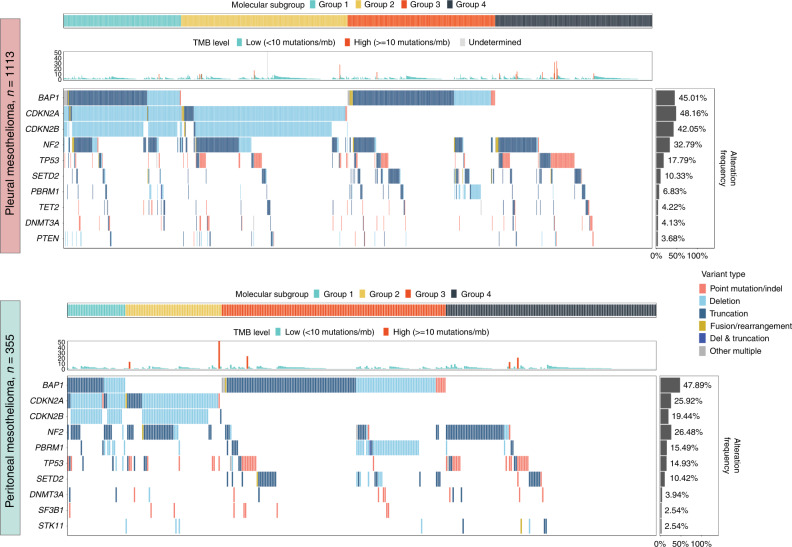
Tiles plot of the most frequent alterations occurring, including TMB values of all the patients in pleural and peritoneal mesothelioma. Four subgroups are detected and visually described with four different colours based on their alterations in *BAP1, CDKN2A* and *CDKN2B*. Group 1 is characterised by alterations in BAP1, *CDKN2A* and *CDKN2B*); Group 2 is characterised by alterations in *CDKN2A* and *CDKN2B*; Group 3 is characterised by alterations in *BAP1* and Group 4 has none.

Based on the most frequently occurring alterations, four distinct subgroups in pleural and peritoneal mesothelioma were identified. In pleural mesothelioma, Group 1 had alterations in *CDKN2A/B* and *BAP1*, Group 2 in *CDKN2A/B* only, Group 3 in *BAP1* only and Group 4 in neither *BAP1* nor *CDKN2A/B* (Fig. 3 and Tables 1 and 2). *NF2* alterations occurred homogeneously in the four groups. This is consistent with the observation of frequent subclonal *NF2* mutations that may occur later in mesothelioma development [16]. Although not investigated, this may correspond to differences in tumour growth seen in mesothelioma mouse models where co-alterations of *BAP1, NF2* and *CDKN2A/B* led to a faster tumour growth compared to tumours with alterations in only one or two genes [17]. In pleural mesothelioma, Group 1 had significantly lower *TP53* alterations (prevalence = 9.0%, *χ*^2^ = 11.7, *P* value = 4.2 × 10^−3^) while Group 4 was characterised by a significantly higher prevalence of *TP53* (prevalence = 30.3%, *χ*^2^ = 31.8, *P* value = 1.5 × 10^−7^) and *RB1* alterations (prevalence = 4.3%, *χ*^2^ = 11.2, *P* value = 5.0 × 10^−3^) compared to the entire pleural mesothelioma group. In addition, more patients exhibited high TMB, consistent with genomically unstable tumours (Tables 1 and 2). *SETD2* (Group 2: prevalence = 3.5%, *χ*^2^ = 11.7, *P* value = 4.2 × 10^−3^, Group 3: prevalence = 22.2%, *χ*^2^ = 42.6, *P* value = 6.6 × 10^−10^) and *PBRM1* (Group 2: prevalence = 0.9%, *χ*^2^ = 15.9, *P* value = 5.0 × 10^−4^, Group 3: prevalence = 16.5%, *χ*^2^ = 40.9, *P* value = 1.5 × 10^−9^) were significantly different compared to the entire pleural mesothelioma cohort (Fig. 2 and Table 1). Similarly, the peritoneal mesothelioma Group 2 (only alterations in *CDKN2A/B* but not in *BAP1*) had a higher prevalence of *NF2* alterations (prevalence = 48.3%, *χ*^2^ = 14.2, *P* value = 1.0 × 10^−3^) compared to the entire cohort of peritoneal cases and Group 3 (only alterations in *BAP1* but not in *CDKN2A/B)* had a lower *NF2* alteration rate (prevalence = 10.4%, *χ*^2^ = 18.0, *P* value = 2.0 × 10^−4^).

**Table 1:** Selected alterations in pleural mesothelioma patients split according to the groups defined in the tiles plot.

Gene	Group 1 (alterations in *CDKN2A/B, BAP1*), *n* = 222	Group 2 (only alterations in *CDKN2A/B),* *n* = 315	Group 3 (only alterations in *BAP1*), *n* = 279	Group 4 (no alterations in *CDKN2A/B, BAP1*), *n* = 297
*NF2*	37.84%	41.27%*	24.37%*	27.95%
*MTAP*	68.04%	62.60%	0.00%	0.85%
*TP53*	9.01%*	16.51%	12.90%	30.30%*
*SETD2*	6.31%	3.49%*	22.22%*	9.43%
*PBRM1*	7.21%	0.95%*	16.49%*	3.70%
*TERT*	1.03%*	15.60%*	3.32%	7.87%
*TET2*	5.86%	3.49%	2.51%	5.39%
*DNMT3A*	4.95%	4.76%	2.51%	4.38%
*PTEN*	4.05%	5.40%	2.15%	3.03%
*BRCA2*	2.70%	2.86%	1.79%	2.69%
*STK11*	2.70%	3.17%	1.08%	1.68%
*PTCH1*	2.25%	1.27%	1.43%	0.67%
*KRAS*	0.45%	3.49%	0.36%	2.36%
*RB1*	0.00%	1.27%	1.08%	4.38%*

Selected genes had a prevalence >1% and can be targeted with available drugs. Chi-square test was used to test for statistically significant; significant values compared to the entire pleural mesothelioma cohort are indicated as *P < 0.05.

**Table 2:** Selected alterations in peritoneal mesothelioma patients split according to the groups defined in the tiles plot.

Gene	Group 1 (alterations in *CDKN2A/B, BAP1*), *n* = 35	Group 2 (only alterations in *CDKN2A/B*), *n* = 58	Group 3 (only alterations in *BAP1*), *n* = 135	Group 4 (no alterations in *CDKN2A/B, BAP1*), *n* = 127
*NF2*	37.14%	48.28%*	10.37%*	30.71%
*MTAP*	72.22%	38.46%	1.64%	1.75%
*TP53*	20.00%	18.97%	10.37%	16.54%
*SETD2*	0.00%	0.00%	20.74%*	7.09%
*PBRM1*	22.86%	1.72%*	31.11%*	3.94%*
*TERT*	3.13%	9.26%	3.31%	4.55%
*TET2*	2.86%	3.45%	1.48%	3.15%
*DNMT3A*	5.71%	3.45%	3.70%	3.94%
*PTEN*	0.00%	1.72%	0.00%	2.36%
*BRCA2*	5.71%	0.00%	2.96%	0.00%
*STK11*	0.00%	5.17%	0.74%	3.94%
*KRAS*	0.00%	5.17%	0.00%	1.57%
*RB1*	0.00%	0.00%	0.74%	3.15%

Selected genes had a prevalence >1% and can be targeted with available drugs. Chi-square test was used to test for statistically significant; significant values compared to the entire pleural mesothelioma cohort are indicated as *P < 0.05.

Due to no available clinical data, we studied the survival, histology and sex priorities of the four different subgroups in the TCGA mesothelioma cohort (*n* = 82) [18, 19]. The subgroups with only *CDKN2A* alteration and the subgroup with *CDKN2A/BAP1* alteration have the worst survival. Interestingly, the subgroup with no *CDKN2A* or *BAP1* alteration survive the longest (Supplementary Fig. 2A). We could not detect differences in histology and sex according to the different alterations (Supplementary Fig. 2B, C).

Mesotheliomas harbour fewer somatic mutations compared to other tumour entities [13] with less than 5% of mesothelioma tumours having a high TMB and an average of 1.7 mutations per Mb [8]. Here, we detected no differences in the TMB between pleural and peritoneal patients (Supplementary Fig. 3) and very few patients with a high TMB (defined as ≥10 mutations/Mb), 15 (1.35%) patients in the pleural and 5 (1.41%) patients in the peritoneal group.

### Alterations in genes of the cell-cycle machinery and its control mechanisms

The most altered gene in pleural mesothelioma is *CDKN2A*, which encodes for inhibitors of the cyclin-dependent kinases 4 and 6 (CDK4/6) involved in cell-cycle regulation [20]. Therefore, *CDKN2A* deletions result in the loss of inhibitory components of the cell-cycle regulation leading to pro-mitotic signals. In this cohort of pleural mesothelioma, 45% of sample harboured copy-number deletions, 1.3% had variants and 1.6% had rearrangements in *CDKN2A*. Compared to pleural mesothelioma, deletions in *CDKN2A/B* were significantly less frequent in peritoneal mesothelioma. Preclinical studies of CDK4/6 inhibitors showed encouraging results in mesothelioma cell lines and xenografts [21], but with limited efficacy in a Phase II clinical trial (NCT02187783).

*CDKN2A* deletions appear often with co-deletions in the adjacent gene *MTAP* (Fig. 1). *MTAP* encodes a protein involved in the adenosine monophosphate and in methionine synthesis and is altered in several malignancies [22]. We detected *CDKN2A* deletions in 48.2% of pleural and in 25.9% of peritoneal mesothelioma. Earlier studies showed a homozygous deletion of *CDKN2A* in 74% of mesothelioma samples and co-deletion of *MTAP* in 91% of these cases [23]. *MTAP* deletions can lead to sensitivity to PRMT5 or MAT2a inhibitors, which showed only limited efficacies in *MTAP*-deficient solid tumours [24].

### Alterations in genes controlling genome integrity

Dysregulated cell cycle, genomic errors and lack of control mechanisms are hallmarks of cancer. *TP53*, tumour suppressor gene is activated upon cell stress and DNA damage and mediates cell-cycle arrest, apoptosis and the transition from S to G1 phase [25]. In previous publications, *TP53* was found to be mutated in 8–19% of mesothelioma tumours [9, 13]. In our study, *TP53* alterations were detected in 17.8% in pleural mesothelioma and in 14.9% of peritoneal mesothelioma patients. A possible therapeutic approach to p53-altered tumours is the use of Hsp90 inhibitors. Hsp90 stabilises mutated p53, which in turn can inhibit the function of wild-type p53 leading to deficient DNA damage response and to sensitisation to chemotherapy [26]. Inhibition of Hsp90 with ganetespib in an unselected patient group lead to partial responses in 52 % of the patients when given in combination with chemotherapy [27].

In this study, several alterations in other genes involved in responses to DNA damage, such as *BAP1*, *PBRM1, BRCA2* and *CHEK2* were detected. BAP1 is a deubiquitinylating enzyme involved in gene transcription, DNA damage repair and cell-cycle control mechanisms [28]. *BAP1* alterations are correlated with environmental carcinogen exposure and *BAP1* knockout mice showed increased incidence of mesothelioma after chronic asbestos exposure [29]. In this cohort, *BAP1* was one of the most frequently altered genes and was found in 45.0% of pleural mesothelioma and 47.9% of peritoneal mesothelioma (Fig. 1 and Supplementary Tables 1–3).

*BRCA1/2* complexes are proteins involved in homologous recombination to suppress genetic instability. Tumours with defects in homologous recombination repair may be susceptible to PARP inhibitors including tumours with *BAP1*-deficiency [30]. In our cohort, *BRCA2* alterations were detected in 2.5% of pleural mesothelioma and in 1.7% of peritoneal mesothelioma tumours, while a recent study detected *BRCA2* alterations in 3 out of 37 pleural mesothelioma patients [31]. A recent Phase IIa clinical trial showed some activity of PARP inhibitors in mesothelioma patients with *BAP1* and *BRCA2* alterations (MiST1 trial) [32].

### Epigenetic modifiers

Mutations occurring in chromatin remodelling complexes are one of the most frequent alterations in different cancers. Genes of the switch/sucrose non-fermentable (SWI/SNF) complex, a tumour suppressor complex, have been previously described to be altered in mesothelioma [33]. Such complex is constituted by 12–15 subunits, including ARID1A, SMARCA4, ARID1B, ARID2, and PBRM1. In pleural and peritoneal mesothelioma, deletions in the *3p21* region, where *PBRM1* is located, were frequently detected. In our cohort, *PBRM1* was altered in 6.8% of pleural mesothelioma and in 15.8% peritoneal tumours. In comparison, in the TCGA dataset (cBioportal), with only pleural mesothelioma cases, 7% of the patients harbour an alteration in the *PBRM1* gene [19]. In recent studies, alterations in *PBRM1* were not detected [34] or overestimated [35] due to the small number of tumours analysed. *PBRM1* alterations are also associated with a less immunogenic tumour microenvironment and resistance to immunotherapy [36]. Moreover, *PBRM1*-defective cancer cells were sensitive to PARP and ATR inhibitors in preclinical models [37]. Thus, further studies are needed to determine the prognostic effect of *PBRM1* alterations in patients receiving immunotherapy and its possible value for patient’s stratification.

Beside its function in chromatin remodelling, the SWI/SNF complex is also a regulator of nucleosome positioning and can regulate gene transcription. The activity of the SWI/SNF complex is opposed by the polycomb repressor complex (PRC), including its subunit enhancer of zeste homologue 2 (EZH2). EZH2 is responsible for trimethylation of the histone H3 at Lys 27 (H3K27me3) and functions as an epigenetic regulator of transcription and is often overexpressed in cancer. Tazemetostat, an EZH2 inhibitor, is currently under evaluation in different Phase I and II clinical trials in tumours with *SMARCB1, SMARCA4* and *EZH2* mutations with positive results in patients with different solid tumours [38].

In our cohort, *SETD2*, a histone methyltransferase, was mutated in 10.3% of pleural and 10.4% of peritoneal mesothelioma tumours. Interestingly, WEE1 inhibitors were shown to selectively target SETD2 mutated cells and are currently in Phase II clinical trials with solid tumours and SETD2 loss [39].

DNMT3A, a histone methyltransferase important for de novo methylation of cytosine residues at CpG sites, is important for regulating gene expression in healthy cells. DNMT3A was shown to be overexpressed in mesothelioma cell lines and its expression correlates with worse prognosis in pleural mesothelioma patients [40]. In our cohort, *DNMT3A* was altered in 4.1% of pleural mesothelioma and in 3.9% of peritoneal mesothelioma cases. Today, DNMT inhibitors such as decitabine and azacytidine, are approved for myelodysplastic syndrome (MDS) and acute myeloid leukaemia and currently under investigation for solid tumours with considerably high toxicity [41].

Similarly, TET2 functions in DNA demethylation and is frequently mutated in haematologic malignancies but seldom in solid tumours [42]. It controls chemokine and PD-L1 expression, lymphocyte infiltration, and cancer immunity [43]. Thus, *TET2* loss might represent a potential a biomarker for predicting the efficacy of response to anti-PD-1/PD-L1 immunotherapy. Here, we detected *TET2* mutations in 4.2% of pleural mesothelioma samples and 2.5% of peritoneal mesothelioma patients. *TET2* alterations were not found in the TCGA dataset [19].

TERT is the catalytic subunit of telomerase and is often reactivated in cancer. In our cohort of pleural and peritoneal mesothelioma patients, the *TERT* promoter was mutated in 7.6% and in 4.7% of the cases, respectively. In mesothelioma, promoter mutations were detected frequently in the non-epithelial subgroup and are associated with a more aggressive disease and with reduced survival in MPM [44].

### Targetable alterations for approved drugs

#### Receptor tyrosine kinases and KRAS

**Fig. 4:**
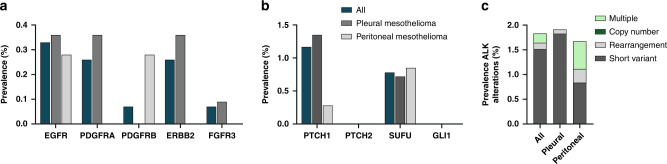
Targetable alterations in the entire cohort. **a** Alterations, which can be targeted with approved drugs, (**b**) alterations in the Hedgehog pathway, (**c**) subtype of alterations in the ALK gene.

We have previously reported the case of a patient with peritoneal mesothelioma harbouring an *ALK* translocation. The patient underwent targeted treatment and achieved a very good partial response [45]. Based on this case, we specifically evaluated the occurrence of *ALK* alterations in this cohort of patients. In 0.36% of pleural mesothelioma patients and 1.13% of peritoneal mesothelioma patients, we detected *ALK* rearrangements and short variant alterations (Fig. 4c). Even if rare, targetable alterations of *KRAS, EGFR, PDGFRA/B, ERBB2* and *FGFR3*, were also detected (Fig. 4a). Specifically, ten patients harboured the G12C mutation in *KRAS*, which could be treated with small molecule inhibitors sotorasib or adagrasib [46, 47] (Supplementary Table 10 and Supplementary Fig. 4).

#### Hedgehog pathway

Alterations in Hedgehog pathway genes, specifically *PTCH1, PTCH2, SUFU, GLI1* were detected. The hedgehog signalling pathway is well conserved and is an important mediator in development, tissue homoeostasis and embryogenesis. Suppressing Hedgehog signalling reduced cell viability in MPM cells [48], and treatment with vismodegib in a rat model of mesothelioma reduced the expression of target genes such as *GLI1, HHIP* and *PTCH1* [49]. Here, we describe alterations in Hedgehog signalling genes *PTCH1* (1.2%) and *SUFU* (0.8%) in the whole mesothelioma cohort (Fig. 4). Even though alterations in the Hedgehog signalling pathway are rare, these can be targeted through commercially available drugs.

#### Hippo pathway

Another gene involved in cell-cycle regulation is the tumour suppressor gene *NF2*, encoding merlin. In our study, *NF2* was altered in 32.8% of pleural mesothelioma and 26.5% of peritoneal mesothelioma cases (Supplementary Table 1). *NF2* can regulate the Hippo pathway via signalling trough YAP and TAZ. Inactivation of *NF2* can lead to hyperactivated YAP and to uncontrolled cell proliferation, thus, YAP inhibitors are currently tested in cell lines and could serve as an option in patients with uncontrolled YAP signalling [50].

NF2 also plays a role in cell-cycle regulation and mTORC1 signalling; inactivation of *NF2* led to PAK1-induced increase of cyclin D1 and consequently to increased cell proliferation [51]. Rapamycin, an mTOR inhibitor, can inhibit proliferation of *NF2*-altered mesothelioma cells in vitro [52]. However, mTOR inhibitors such as everolimus had limited clinical activity in a Phase II clinical trial in an unselected mesothelioma cohort [53]. Currently, second and third-generation mTOR inhibitors and PI3K/mTOR inhibitors are under investigation in other malignancies [54].

## Discussion

This is the largest analysis formed on mesothelioma samples profiled by NGS so far. Thank to this broad number of cases we could identify four subtypes of mesothelioma according to their molecular genetic alterations in *CDKN2A, CDKN2B* and *BAP1*. We also detected the occurrence of rare genomic alterations, which can be targeted with FDA-approved or experimental drugs. Indeed, for several tumour types, patient stratification allows personalised treatment approach and improved survival.

Currently, mesothelioma patients are classified according to their histological subtype, however, due to major differences in their genomic features, genomic profiling may add value for treatment stratification and might refine future treatment strategies. Moreover, genomic subgroups might be due to different tumorigenesis mechanisms as already described in breast cancer, where oncogenic alterations lead to different tumour types with different clinical outcomes (reviewed in ref. [55]). Hmeljak et al. performed clustering of molecular data using two different algorithms and defined four different subgroups with different survival properties [9]. In our study, we identified four distinct subgroups of patients based on their genomic background (Fig. 3 and Tables 1 and 2). Those different genomic subgroups might have different clinical characteristics and response to treatment, and this finding warrants further investigations. Preclinical models of mesothelioma confirm that specific alterations can lead to different outcomes, for example, *CDKN2A* loss leads to poor outcomes [56] and *BAP1* alterations in combination with *NF2* and *CDKN2A/B* to rapid disease onset [17].

We defined four different groups with significantly different prevalence of alteration in *BAP1* and *CDKN2A* genes. For example, alterations in *PBRM1* and *SETD2* were mainly associated to Group 3 (*BAP1* alteration only), while *TP53* alterations occurred mainly in Group 4 (no *CDKN2A/BAP1* alteration) in pleural mesothelioma. Patients with a combined loss of *TP53* and *RB1* might even have worse prognoses as described in different malignancies [57, 58]. Interestingly, the patients in Group 4 had a worse survival in the TCGA cohort compared to Group 3 with only *BAP1* alteration, which might be due to the significantly higher mutation rate in *TP53* and *RB1* (Supplementary Fig. 2). However, further studies are needed to understand the differences in therapy response in these molecular subgroups.

The most common alterations occurred in *BAP1*, *NF2* or deletions in *CDKN2A/B*, in line with the Catalogue of Somatic Mutations in Cancer (COSMIC) database [59] and in cBioportal [18]. Compared to previously reported data, we detected a much higher frequency of *BAP1*, *CDKN2A* and *NF2* alterations in our cohort and this might be due to a larger cohort size. In our cohort, *NF2* is altered in 32.8% of pleural and 26.5% of peritoneal mesothelioma, compared to other studies with alterations in about 20% of the cases [13, 31, 34, 60]. Similarly, *BAP1* alterations were more frequently detected (45.0% pleural and 47.9% peritoneal mesothelioma), compared to the studies from Bueno et al. and Quetel et al. (with 23% and 24.4%, respectively) [13, 60]. However, this prevalence is consistent with what has been recently observed in low-risk mesothelioma patients [15]. Interestingly, deletions in *CDKN2A/B* occur more frequently in pleural compared to peritoneal mesothelioma, as previously described [61, 62]. A possible explanation is that chromosome arm deletions are associated with asbestos exposure and, therefore, a possible different causality for peritoneal compared to pleural mesothelioma. Few cases showed an increased TMB, therefore it is not possible to discriminate if co-occurring alterations were truly enriched and functionally important, rather than just passenger by-products of any mutated gene.

In addition, this analysis revealed alterations in 19 genes with a prevalence higher than 2%. Moreover, co-alterations showed the clustering of different groups of patients with mutations in *BAP1, PBRM1, SETD2*; or in *MYC, TERT, RB1* and *TP53* or in *CDKN2A/B, NF2* and *BAP1*. Despite the occurrence of alterations in several genes and co-mutations, cases with increased tumour mutational burden were rare, but leave the option for immunotherapy with immune checkpoint inhibitors in some patients. A broad analysis of the mutation spectra might become a stratification factor for patients receiving immunotherapy. Analysing mutations in genes correlated with response to treatment is of major importance for patient selection. In lung cancer, patients with *STK11* mutations showed resistance to anti-PD-1 inhibitors [63], and deletions in *CDKN2A* were negatively correlated with T-cell markers in different cancer types [64]. These findings warrant further investigations about differences in tumour development and possibly responses to treatment.

Based on our data, targeted treatments might also become a possible approach for mesothelioma, as alterations in hedgehog pathway-related genes (e.g., *PTCH1/2* and *SUFU*) and hippo pathway-related genes (particularly *NF2*) were found. Moreover, we detected alterations in *KRAS, EGFR, PDGFRA/B, ERBB2, FGFR3* and *ALK*. Even if those alterations are rare, their detection and the use of targeted treatments can change patient outcomes. We have recently shown that targeting a *STRN-ALK* rearrangement with an ALK-inhibitor in a patient with peritoneal mesothelioma, could lead to prolonged response [45] and Popat et al. presented a case with *PTCH1* alteration where vismodegib treatment led to good response [65].

A major limitation of our study is the lack of matching clinical data about histology, outcome, treatment allocation and response to treatment; this was not possible due to the retrospective nature of this analysis. Nevertheless, clinical data from the TCGA cohort show significant differences in overall survival between the four defined groups but no differences in sex and histology. These findings might help understand mechanisms related to tumour development and provide novel insights into genomic subgroups for patient stratification for clinical trials. A recently opened clinical trial (MiST) is the first to stratify patients according to their molecular alterations, where *BRCA1* mutated/*BAP1* negative patients receive PARP inhibitors, while *CDKN2A* negative patients a CDK4/6 inhibitor or immunotherapy depending on their PD-L1 expression. However, in this study, the specified molecular alterations were measured by IHC assays, no NGS was performed for patient stratification, which might open the possibility of more tailored treatment approaches. In addition, the advantage of NGS lays in its cost-effectiveness, in delivering reliable results without interpersonal differences for interpretation, and it can save material compared to multi-step testing strategies.

## Conclusion

Precision medicine, including high-throughput genomic screening, has tremendously improved the outcome of patients with a high prevalence of mutations, in particular in patients with lung cancer, breast cancer and melanoma. Nevertheless, also in rare malignancies and in malignancies with low numbers of somatic mutations, targetable mutations need to be analysed in order to enlarge treatment options for difficult-to-treat cancers. Our results indicate, that molecular analysis for mesothelioma should be implemented.

## Supplementary information

Supplementary Information

Reproducibility checklist

## Data Availability

All relevant data are provided in the manuscript. Due to HIPAA requirements, FMI is not consented to share individualised patient genomic data, which contains potentially identifying or sensitive patient information. FMI is committed to collaborative data analysis, and we have well-established, and widely utilised mechanisms by which investigators can query our core genomic database of >500,000 de-identified sequenced cancers to obtain aggregated datasets.
