# Supplementary material for: Genomic landscape of pleural and peritoneal mesothelioma tumours
Source: Br J Cancer. 2022 Sep 22;127(11):1997–2005. doi: 10.1038/s41416-022-01979-0 (PMC9681755; doi:10.1038/s41416-022-01979-0)
Supplement: Supplementary file 1 — Supplementary Information [file 41416_2022_1979_MOESM1_ESM.docx]

**Supplementary Material**

**Genomic landscape of pleural and peritoneal mesothelioma tumors**

| **A**  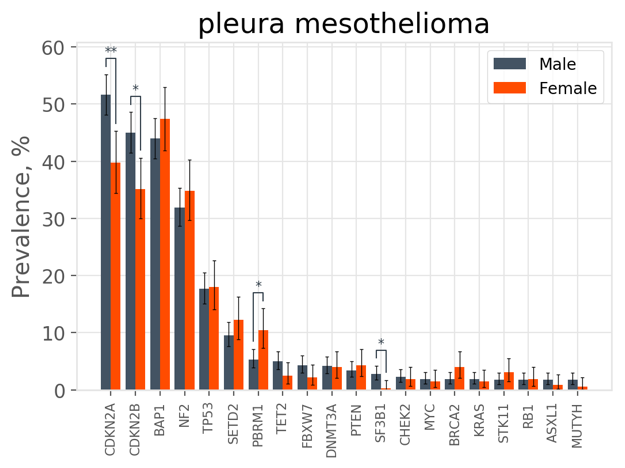 | **B**  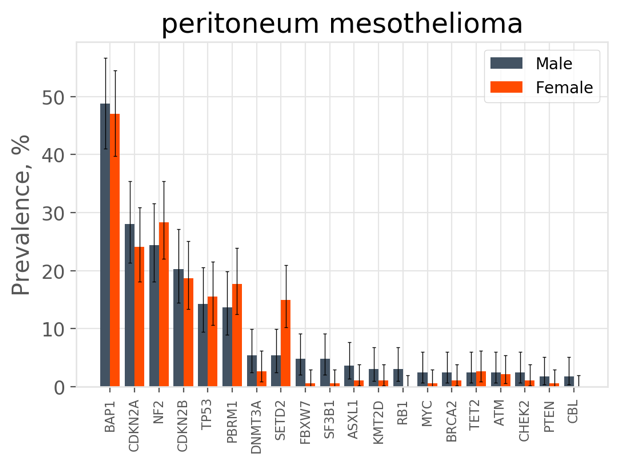 |
| --- | --- |

**Supplementary Figure 1.** Sex differences in genetic alterations in A) pleural mesothelioma and B) peritoneal mesothelioma. Error bars indicate 95% confidence interval (binomial) and statistical significance was tested by fisher’s exact and is indicated as * p < 0.05, ** *p* < 0.01.

**
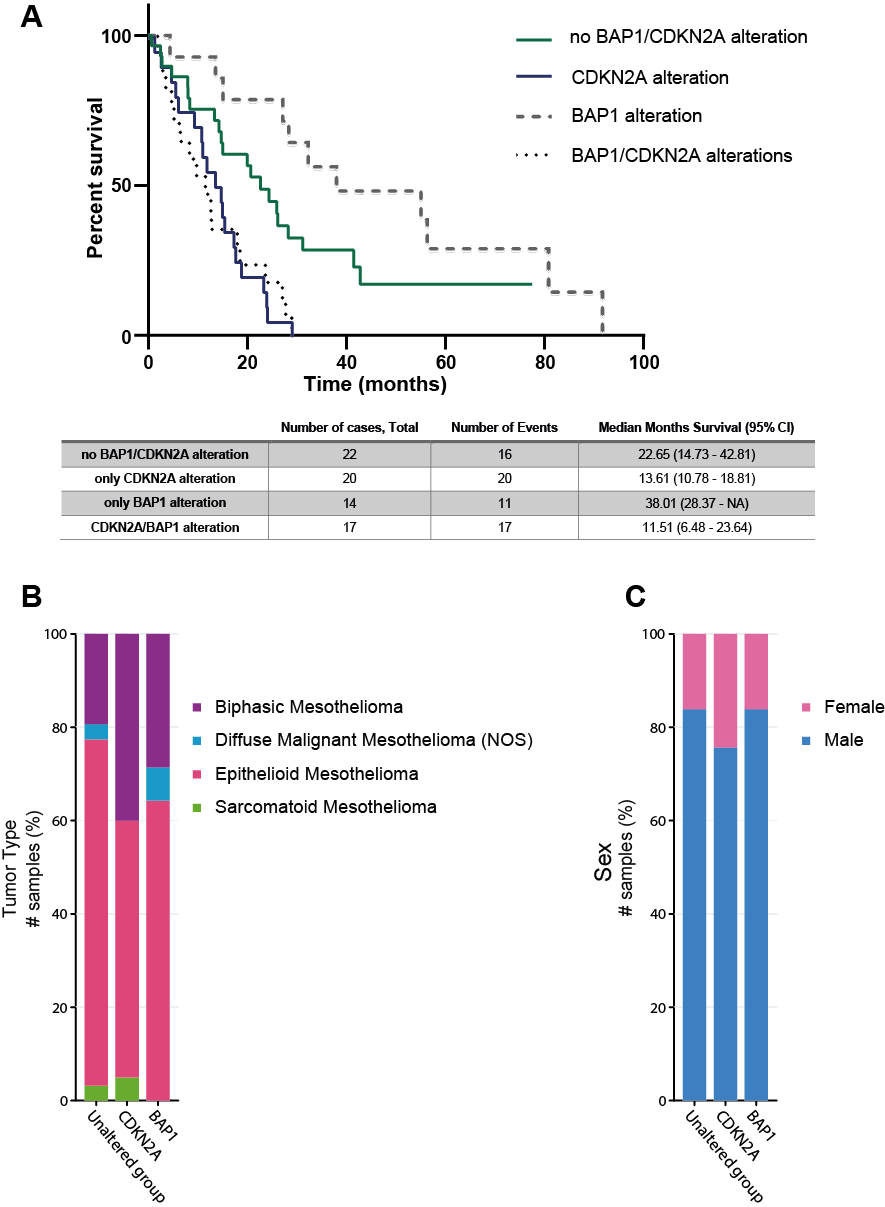
**

**Supplementary Figure 2.** Clinical data from the TCGA mesothelioma cohort (n = 82). A) Kaplan-Meier estimates of overall survival of the patient group without any *BAP1* or *CDKN2A* alteration (green solid line), with *CDKN2A* alteration only (blue solid line), with *BAP1* alteration only (grey dotted line), and with *BAP1* and *CDKN2A* alteration (black dotted line), Logrank test p-value = 8.601 *10^-6^, B) distribution of the histological subtypes in the unaltered patient group, in the *CDKN2A* altered patient group and in the *BAP1* altered patient group, C) sex distribution in the unaltered patient group, in the *CDKN2A* altered patient group and in the *BAP1* altered patient group.


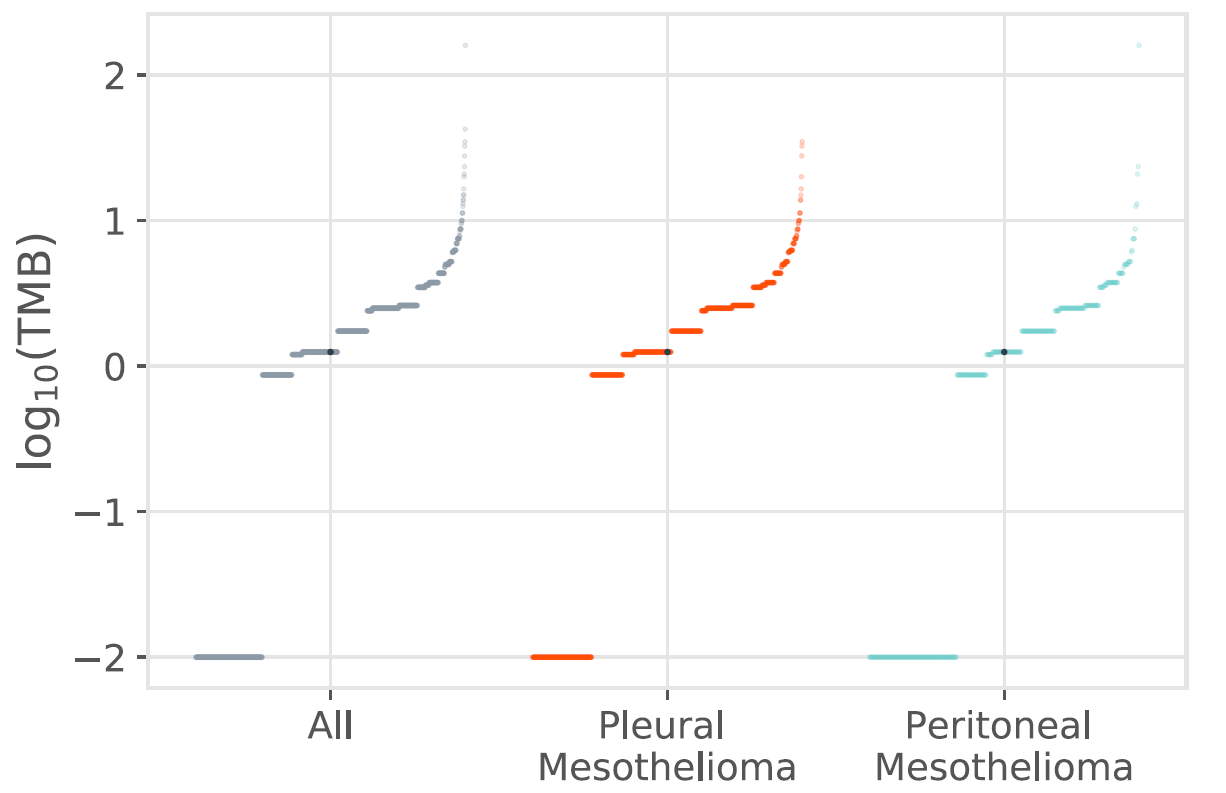


**Supplementary Figure 3.** Overview on tumor mutational burden of pleural and peritoneal mesothelioma patients


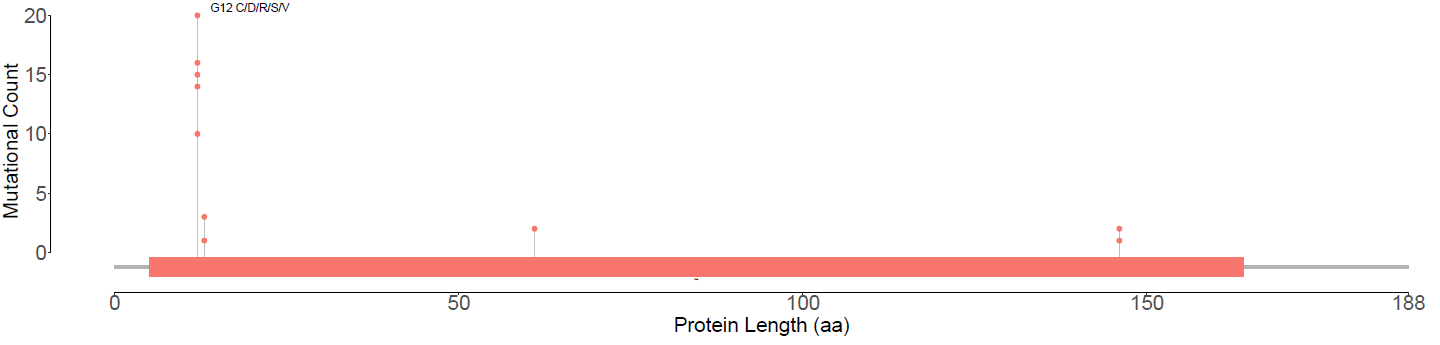


**Supplementary Figure 4.** Graphical overview on detected *KRAS* alterations in both groups.

**Supplementary Table 1.** Prevalence of genetic alterations in all mesothelioma cases

|  | **# Assessable**  **Samples** | **# Variant Samples** | **Prevalence  (% variant samples)** | **Prevalence  (Lower 95% CI)** | **Prevalence  (Upper 95% CI)** | **Short Variant** | **Rearrangement** | **Amplification (CN)** | **Deletion (CN)** | **Multiple** |
| --- | --- | --- | --- | --- | --- | --- | --- | --- | --- | --- |
| **BAP1** | 1536 | 693 | 45.12% | 42.61% | 47.65% | 27.47% | 3.97% | 0.00% | 13.09% | 0.59% |
| **CDKN2A** | 1536 | 648 | 42.19% | 39.70% | 44.70% | 1.69% | 1.63% | 0.00% | 38.67% | 0.20% |
| **CDKN2B** | 1536 | 553 | 36.00% | 33.60% | 38.46% | 0.00% | 0.52% | 0.00% | 35.48% | 0.00% |
| **NF2** | 1536 | 481 | 31.32% | 29.00% | 33.70% | 25.39% | 1.37% | 0.00% | 4.30% | 0.26% |
| **MTAP** | 679 | 185 | 27.25% | 23.93% | 30.76% | 0.00% | 0.29% | 0.00% | 26.95% | 0.00% |
| **TP53** | 1536 | 265 | 17.25% | 15.39% | 19.24% | 16.15% | 0.33% | 0.00% | 0.65% | 0.13% |
| **SETD2** | 1536 | 157 | 10.22% | 8.75% | 11.85% | 7.81% | 0.91% | 0.00% | 1.43% | 0.07% |
| **PBRM1** | 1536 | 139 | 9.05% | 7.66% | 10.60% | 2.34% | 0.85% | 0.00% | 5.73% | 0.13% |
| **TERT** | 1365 | 95 | 6.96% | 5.67% | 8.44% | 6.96% | 0.00% | 0.00% | 0.00% | 0.00% |
| **DNMT3A** | 1536 | 66 | 4.30% | 3.34% | 5.43% | 3.97% | 0.26% | 0.00% | 0.07% | 0.00% |
| **TET2** | 1536 | 58 | 3.78% | 2.88% | 4.85% | 3.78% | 0.00% | 0.00% | 0.00% | 0.00% |
| **FBXW7** | 1536 | 52 | 3.39% | 2.54% | 4.42% | 2.21% | 0.33% | 0.00% | 0.85% | 0.00% |
| **PTEN** | 1536 | 46 | 2.99% | 2.20% | 3.97% | 1.69% | 0.00% | 0.00% | 1.30% | 0.00% |
| **BRCA2** | 1536 | 36 | 2.34% | 1.65% | 3.23% | 1.95% | 0.13% | 0.00% | 0.26% | 0.00% |
| **STK11** | 1536 | 36 | 2.34% | 1.65% | 3.23% | 1.11% | 0.33% | 0.00% | 0.85% | 0.07% |
| **RAD21** | 679 | 15 | 2.21% | 1.24% | 3.62% | 0.00% | 0.00% | 2.21% | 0.00% | 0.00% |
| **SF3B1** | 1536 | 32 | 2.08% | 1.43% | 2.93% | 2.08% | 0.00% | 0.00% | 0.00% | 0.00% |
| **KMT2C** | 686 | 14 | 2.04% | 1.12% | 3.40% | 1.75% | 0.15% | 0.00% | 0.00% | 0.15% |
| **CHEK2** | 1536 | 31 | 2.02% | 1.38% | 2.85% | 1.76% | 0.07% | 0.00% | 0.20% | 0.00% |
| **RB1** | 1536 | 29 | 1.89% | 1.27% | 2.70% | 0.98% | 0.07% | 0.00% | 0.85% | 0.00% |
| **ARID2** | 857 | 16 | 1.87% | 1.07% | 3.01% | 1.05% | 0.35% | 0.00% | 0.47% | 0.00% |
| **MYC** | 1536 | 27 | 1.76% | 1.16% | 2.55% | 0.07% | 0.13% | 1.56% | 0.00% | 0.00% |
| **KRAS** | 1536 | 27 | 1.76% | 1.16% | 2.55% | 1.30% | 0.00% | 0.00% | 0.00% | 0.46% |
| **ASXL1** | 1536 | 26 | 1.69% | 1.11% | 2.47% | 1.56% | 0.13% | 0.00% | 0.00% | 0.00% |
| **MUTYH** | 1536 | 23 | 1.50% | 0.95% | 2.24% | 1.50% | 0.00% | 0.00% | 0.00% | 0.00% |
| **CTNNB1** | 1536 | 22 | 1.43% | 0.90% | 2.16% | 1.11% | 0.13% | 0.00% | 0.20% | 0.00% |
| **ATM** | 1536 | 21 | 1.37% | 0.85% | 2.08% | 1.17% | 0.20% | 0.00% | 0.00% | 0.00% |
| **RICTOR** | 1536 | 20 | 1.30% | 0.80% | 2.00% | 0.00% | 0.00% | 1.30% | 0.00% | 0.00% |
| **CREBBP** | 1536 | 19 | 1.24% | 0.75% | 1.92% | 0.91% | 0.33% | 0.00% | 0.00% | 0.00% |
| **KMT2D** | 1536 | 19 | 1.24% | 0.75% | 1.92% | 1.11% | 0.13% | 0.00% | 0.00% | 0.00% |
| **PTCH1** | 1536 | 18 | 1.17% | 0.70% | 1.85% | 0.78% | 0.26% | 0.00% | 0.13% | 0.00% |
| **LRP1B** | 857 | 10 | 1.17% | 0.56% | 2.14% | 0.70% | 0.35% | 0.00% | 0.12% | 0.00% |
| **SPTA1** | 686 | 8 | 1.17% | 0.50% | 2.28% | 1.17% | 0.00% | 0.00% | 0.00% | 0.00% |
| **ARID1A** | 1536 | 17 | 1.11% | 0.65% | 1.77% | 1.04% | 0.00% | 0.00% | 0.07% | 0.00% |
| **EPHA3** | 1536 | 16 | 1.04% | 0.60% | 1.69% | 0.13% | 0.00% | 0.91% | 0.00% | 0.00% |
| **LZTR1** | 686 | 7 | 1.02% | 0.41% | 2.09% | 0.87% | 0.15% | 0.00% | 0.00% | 0.00% |
| **SUFU** | 1536 | 12 | 0.78% | 0.40% | 1.36% | 0.39% | 0.00% | 0.00% | 0.39% | 0.00% |
| **ALK** | 1536 | 9 | 0.59% | 0.27% | 1.11% | 0.33% | 0.26% | 0.00% | 0.00% | 0.00% |
| **EGFR** | 1536 | 5 | 0.33% | 0.11% | 0.76% | 0.20% | 0.00% | 0.13% | 0.00% | 0.00% |
| **PDGFRA** | 1536 | 4 | 0.26% | 0.07% | 0.67% | 0.07% | 0.00% | 0.20% | 0.00% | 0.00% |
| **ERBB2** | 1536 | 4 | 0.26% | 0.07% | 0.67% | 0.13% | 0.00% | 0.13% | 0.00% | 0.00% |
| **PDGFRB** | 1536 | 1 | 0.07% | 0.00% | 0.36% | 0.07% | 0.00% | 0.00% | 0.00% | 0.00% |
| **FGFR3** | 1536 | 1 | 0.07% | 0.00% | 0.36% | 0.00% | 0.07% | 0.00% | 0.00% | 0.00% |
| **PTCH2** | 686 | 0 | 0.00% | 0.00% | 0.54% | 0.00% | 0.00% | 0.00% | 0.00% | 0.00% |
| **SOX2** | 1536 | 0 | 0.00% | 0.00% | 0.24% | 0.00% | 0.00% | 0.00% | 0.00% | 0.00% |
| **CSF1R** | 1536 | 0 | 0.00% | 0.00% | 0.24% | 0.00% | 0.00% | 0.00% | 0.00% | 0.00% |
| **GLI1** | 686 | 0 | 0.00% | 0.00% | 0.54% | 0.00% | 0.00% | 0.00% | 0.00% | 0.00% |

**Table 2:** Prevalence of genetic alterations in pleural mesothelioma cases

|  | **# Assessable Samples** | **# Variant Samples** | **Prevalence (% variant samples)** | **Prevalence (Lower 95% CI)** | **Prevalence (Upper 95% CI)** | **Short Variant** | **Rearrangement** | **Amplification (CN)** | **Deletion (CN)** | **Multiple** |
| --- | --- | --- | --- | --- | --- | --- | --- | --- | --- | --- |
| **CDKN2A** | 1113 | 536 | 48.16% | 45.19% | 51.14% | 1.26% | 1.62% | 0.00% | 45.01% | 0.27% |
| **BAP1** | 1113 | 501 | 45.01% | 42.06% | 47.99% | 28.39% | 4.22% | 0.00% | 11.86% | 0.54% |
| **CDKN2B** | 1113 | 469 | 42.14% | 39.22% | 45.10% | 0.00% | 0.63% | 0.00% | 41.51% | 0.00% |
| **NF2** | 1113 | 365 | 32.79% | 30.04% | 35.64% | 25.97% | 1.53% | 0.00% | 4.94% | 0.36% |
| **MTAP** | 461 | 149 | 32.32% | 28.07% | 36.80% | 0.00% | 0.00% | 0.00% | 32.32% | 0.00% |
| **TP53** | 1113 | 198 | 17.79% | 15.59% | 20.17% | 16.53% | 0.45% | 0.00% | 0.63% | 0.18% |
| **SETD2** | 1113 | 115 | 10.33% | 8.61% | 12.27% | 8.09% | 0.81% | 0.00% | 1.35% | 0.09% |
| **TERT** | 984 | 75 | 7.62% | 6.04% | 9.46% | 7.62% | 0.00% | 0.00% | 0.00% | 0.00% |
| **PBRM1** | 1113 | 76 | 6.83% | 5.42% | 8.47% | 2.34% | 0.72% | 0.00% | 3.68% | 0.09% |
| **TET2** | 1113 | 47 | 4.22% | 3.12% | 5.58% | 4.22% | 0.00% | 0.00% | 0.00% | 0.00% |
| **DNMT3A** | 1113 | 46 | 4.13% | 3.04% | 5.47% | 3.86% | 0.27% | 0.00% | 0.00% | 0.00% |
| **FBXW7** | 1113 | 41 | 3.68% | 2.66% | 4.96% | 2.43% | 0.36% | 0.00% | 0.90% | 0.00% |
| **PTEN** | 1113 | 41 | 3.68% | 2.66% | 4.96% | 1.98% | 0.00% | 0.00% | 1.71% | 0.00% |
| **BRCA2** | 1113 | 28 | 2.52% | 1.68% | 3.62% | 1.98% | 0.18% | 0.00% | 0.36% | 0.00% |
| **ARID2** | 652 | 16 | 2.45% | 1.41% | 3.95% | 1.38% | 0.46% | 0.00% | 0.61% | 0.00% |
| **RAD21** | 461 | 10 | 2.17% | 1.04% | 3.95% | 0.00% | 0.00% | 2.17% | 0.00% | 0.00% |
| **STK11** | 1113 | 24 | 2.16% | 1.39% | 3.19% | 1.17% | 0.36% | 0.00% | 0.54% | 0.09% |
| **CHEK2** | 1113 | 24 | 2.16% | 1.39% | 3.19% | 1.89% | 0.09% | 0.00% | 0.18% | 0.00% |
| **SF3B1** | 1113 | 23 | 2.07% | 1.31% | 3.08% | 2.07% | 0.00% | 0.00% | 0.00% | 0.00% |
| **KMT2C** | 523 | 10 | 1.91% | 0.92% | 3.49% | 1.53% | 0.19% | 0.00% | 0.00% | 0.19% |
| **KRAS** | 1113 | 20 | 1.80% | 1.10% | 2.76% | 1.35% | 0.00% | 0.00% | 0.00% | 0.45% |
| **MYC** | 1113 | 20 | 1.80% | 1.10% | 2.76% | 0.00% | 0.09% | 1.71% | 0.00% | 0.00% |
| **RB1** | 1113 | 20 | 1.80% | 1.10% | 2.76% | 1.08% | 0.00% | 0.00% | 0.72% | 0.00% |
| **ASXL1** | 1113 | 17 | 1.53% | 0.89% | 2.43% | 1.35% | 0.18% | 0.00% | 0.00% | 0.00% |
| **RICTOR** | 1113 | 17 | 1.53% | 0.89% | 2.43% | 0.00% | 0.00% | 1.53% | 0.00% | 0.00% |
| **MUTYH** | 1113 | 16 | 1.44% | 0.82% | 2.32% | 1.44% | 0.00% | 0.00% | 0.00% | 0.00% |
| **PTCH1** | 1113 | 15 | 1.35% | 0.76% | 2.21% | 0.90% | 0.27% | 0.00% | 0.18% | 0.00% |
| **LRP1B** | 652 | 8 | 1.23% | 0.53% | 2.40% | 0.77% | 0.31% | 0.00% | 0.15% | 0.00% |
| **CREBBP** | 1113 | 13 | 1.17% | 0.62% | 1.99% | 0.99% | 0.18% | 0.00% | 0.00% | 0.00% |
| **CTNNB1** | 1113 | 13 | 1.17% | 0.62% | 1.99% | 0.81% | 0.18% | 0.00% | 0.18% | 0.00% |
| **NSD3** | 461 | 5 | 1.08% | 0.35% | 2.51% | 0.00% | 0.00% | 1.08% | 0.00% | 0.00% |
| **EPHA3** | 1113 | 12 | 1.08% | 0.56% | 1.88% | 0.18% | 0.00% | 0.90% | 0.00% | 0.00% |
| **ATM** | 1113 | 12 | 1.08% | 0.56% | 1.88% | 0.81% | 0.27% | 0.00% | 0.00% | 0.00% |
| **SUFU** | 1113 | 8 | 0.72% | 0.31% | 1.41% | 0.45% | 0.00% | 0.00% | 0.27% | 0.00% |
| **ERBB2** | 1113 | 4 | 0.36% | 0.10% | 0.92% | 0.18% | 0.00% | 0.18% | 0.00% | 0.00% |
| **EGFR** | 1113 | 4 | 0.36% | 0.10% | 0.92% | 0.18% | 0.00% | 0.18% | 0.00% | 0.00% |
| **PDGFRA** | 1113 | 4 | 0.36% | 0.10% | 0.92% | 0.09% | 0.00% | 0.27% | 0.00% | 0.00% |
| **ALK** | 1113 | 4 | 0.36% | 0.10% | 0.92% | 0.36% | 0.00% | 0.00% | 0.00% | 0.00% |
| **FGFR3** | 1113 | 1 | 0.09% | 0.00% | 0.50% | 0.00% | 0.09% | 0.00% | 0.00% | 0.00% |
| **SOX2** | 1113 | 0 | 0.00% | 0.00% | 0.33% | 0.00% | 0.00% | 0.00% | 0.00% | 0.00% |
| **PTCH2** | 523 | 0 | 0.00% | 0.00% | 0.70% | 0.00% | 0.00% | 0.00% | 0.00% | 0.00% |
| **CSF1R** | 1113 | 0 | 0.00% | 0.00% | 0.33% | 0.00% | 0.00% | 0.00% | 0.00% | 0.00% |
| **PDGFRB** | 1113 | 0 | 0.00% | 0.00% | 0.33% | 0.00% | 0.00% | 0.00% | 0.00% | 0.00% |
| **GLI1** | 523 | 0 | 0.00% | 0.00% | 0.70% | 0.00% | 0.00% | 0.00% | 0.00% | 0.00% |

**Supplementary Table 3:** Prevalence of genetic alterations in peritoneal mesothelioma cases

|  | **# Assessable Samples** | **# Variant Samples** | **Prevalence (% variant samples)** | **Prevalence (Lower 95% CI)** | **Prevalence (Upper 95% CI)** | **Short Variant** | **Rearrangement** | **Amplification (CN)** | **Deletion (CN)** | **Multiple** |
| --- | --- | --- | --- | --- | --- | --- | --- | --- | --- | --- |
| **BAP1** | 355 | 170 | 47.89% | 42.59% | 53.22% | 26.76% | 3.38% | 0.00% | 17.18% | 0.56% |
| **NF2** | 355 | 94 | 26.48% | 21.96% | 31.39% | 22.82% | 1.13% | 0.00% | 2.54% | 0.00% |
| **CDKN2A** | 355 | 92 | 25.92% | 21.43% | 30.80% | 2.82% | 1.69% | 0.00% | 21.41% | 0.00% |
| **CDKN2B** | 355 | 69 | 19.44% | 15.45% | 23.94% | 0.00% | 0.28% | 0.00% | 19.15% | 0.00% |
| **PBRM1** | 355 | 56 | 15.77% | 12.14% | 19.99% | 1.97% | 1.13% | 0.00% | 12.39% | 0.28% |
| **MTAP** | 162 | 25 | 15.43% | 10.24% | 21.93% | 0.00% | 1.23% | 0.00% | 14.20% | 0.00% |
| **TP53** | 355 | 53 | 14.93% | 11.39% | 19.07% | 14.08% | 0.00% | 0.00% | 0.85% | 0.00% |
| **SETD2** | 355 | 37 | 10.42% | 7.45% | 14.08% | 7.61% | 1.13% | 0.00% | 1.69% | 0.00% |
| **TERT** | 317 | 15 | 4.73% | 2.67% | 7.68% | 4.73% | 0.00% | 0.00% | 0.00% | 0.00% |
| **DNMT3A** | 355 | 14 | 3.94% | 2.17% | 6.53% | 3.38% | 0.28% | 0.00% | 0.28% | 0.00% |
| **KMT2C** | 155 | 4 | 2.58% | 0.71% | 6.48% | 2.58% | 0.00% | 0.00% | 0.00% | 0.00% |
| **SF3B1** | 355 | 9 | 2.54% | 1.17% | 4.76% | 2.54% | 0.00% | 0.00% | 0.00% | 0.00% |
| **FBXW7** | 355 | 9 | 2.54% | 1.17% | 4.76% | 1.41% | 0.28% | 0.00% | 0.85% | 0.00% |
| **TET2** | 355 | 9 | 2.54% | 1.17% | 4.76% | 2.54% | 0.00% | 0.00% | 0.00% | 0.00% |
| **STK11** | 355 | 9 | 2.54% | 1.17% | 4.76% | 0.85% | 0.28% | 0.00% | 1.41% | 0.00% |
| **ASXL1** | 355 | 8 | 2.25% | 0.98% | 4.39% | 2.25% | 0.00% | 0.00% | 0.00% | 0.00% |
| **ATM** | 355 | 8 | 2.25% | 0.98% | 4.39% | 2.25% | 0.00% | 0.00% | 0.00% | 0.00% |
| **CTNNB1** | 355 | 7 | 1.97% | 0.80% | 4.02% | 1.69% | 0.00% | 0.00% | 0.28% | 0.00% |
| **KMT2D** | 355 | 7 | 1.97% | 0.80% | 4.02% | 1.97% | 0.00% | 0.00% | 0.00% | 0.00% |
| **MUTYH** | 355 | 7 | 1.97% | 0.80% | 4.02% | 1.97% | 0.00% | 0.00% | 0.00% | 0.00% |
| **LZTR1** | 155 | 3 | 1.94% | 0.40% | 5.55% | 1.94% | 0.00% | 0.00% | 0.00% | 0.00% |
| **SPTA1** | 155 | 3 | 1.94% | 0.40% | 5.55% | 1.94% | 0.00% | 0.00% | 0.00% | 0.00% |
| **CHEK2** | 355 | 6 | 1.69% | 0.62% | 3.64% | 1.41% | 0.00% | 0.00% | 0.28% | 0.00% |
| **ARID1A** | 355 | 6 | 1.69% | 0.62% | 3.64% | 1.41% | 0.00% | 0.00% | 0.28% | 0.00% |
| **BRCA2** | 355 | 6 | 1.69% | 0.62% | 3.64% | 1.69% | 0.00% | 0.00% | 0.00% | 0.00% |
| **CREBBP** | 355 | 6 | 1.69% | 0.62% | 3.64% | 0.85% | 0.85% | 0.00% | 0.00% | 0.00% |
| **MYC** | 355 | 5 | 1.41% | 0.46% | 3.26% | 0.00% | 0.28% | 1.13% | 0.00% | 0.00% |
| **RB1** | 355 | 5 | 1.41% | 0.46% | 3.26% | 0.85% | 0.28% | 0.00% | 0.28% | 0.00% |
| **KRAS** | 355 | 5 | 1.41% | 0.46% | 3.26% | 1.13% | 0.00% | 0.00% | 0.00% | 0.28% |
| **FAT1** | 155 | 2 | 1.29% | 0.16% | 4.58% | 0.65% | 0.00% | 0.00% | 0.00% | 0.65% |
| **FOXP1** | 155 | 2 | 1.29% | 0.16% | 4.58% | 0.65% | 0.00% | 0.00% | 0.65% | 0.00% |
| **CSF3R** | 162 | 2 | 1.23% | 0.15% | 4.39% | 1.23% | 0.00% | 0.00% | 0.00% | 0.00% |
| **RAD21** | 162 | 2 | 1.23% | 0.15% | 4.39% | 0.00% | 0.00% | 1.23% | 0.00% | 0.00% |
| **PTEN** | 355 | 4 | 1.13% | 0.31% | 2.86% | 1.13% | 0.00% | 0.00% | 0.00% | 0.00% |
| **PIK3CA** | 355 | 4 | 1.13% | 0.31% | 2.86% | 1.13% | 0.00% | 0.00% | 0.00% | 0.00% |
| **CIC** | 355 | 4 | 1.13% | 0.31% | 2.86% | 0.56% | 0.00% | 0.00% | 0.56% | 0.00% |
| **ALK** | 355 | 4 | 1.13% | 0.31% | 2.86% | 0.28% | 0.85% | 0.00% | 0.00% | 0.00% |
| **GRIN2A** | 193 | 2 | 1.04% | 0.13% | 3.69% | 1.04% | 0.00% | 0.00% | 0.00% | 0.00% |
| **NOTCH4** | 193 | 2 | 1.04% | 0.13% | 3.69% | 1.04% | 0.00% | 0.00% | 0.00% | 0.00% |
| **LRP1B** | 193 | 2 | 1.04% | 0.13% | 3.69% | 0.52% | 0.52% | 0.00% | 0.00% | 0.00% |
| **SUFU** | 355 | 3 | 0.85% | 0.17% | 2.45% | 0.28% | 0.00% | 0.00% | 0.56% | 0.00% |
| **PTCH1** | 355 | 1 | 0.28% | 0.01% | 1.56% | 0.28% | 0.00% | 0.00% | 0.00% | 0.00% |
| **PDGFRB** | 355 | 1 | 0.28% | 0.01% | 1.56% | 0.28% | 0.00% | 0.00% | 0.00% | 0.00% |
| **EGFR** | 355 | 1 | 0.28% | 0.01% | 1.56% | 0.28% | 0.00% | 0.00% | 0.00% | 0.00% |
| **PDGFRA** | 355 | 0 | 0.00% | 0.00% | 1.03% | 0.00% | 0.00% | 0.00% | 0.00% | 0.00% |
| **SOX2** | 355 | 0 | 0.00% | 0.00% | 1.03% | 0.00% | 0.00% | 0.00% | 0.00% | 0.00% |
| **PTCH2** | 155 | 0 | 0.00% | 0.00% | 2.35% | 0.00% | 0.00% | 0.00% | 0.00% | 0.00% |
| **CSF1R** | 355 | 0 | 0.00% | 0.00% | 1.03% | 0.00% | 0.00% | 0.00% | 0.00% | 0.00% |
| **ERBB2** | 355 | 0 | 0.00% | 0.00% | 1.03% | 0.00% | 0.00% | 0.00% | 0.00% | 0.00% |
| **FGFR3** | 355 | 0 | 0.00% | 0.00% | 1.03% | 0.00% | 0.00% | 0.00% | 0.00% | 0.00% |
| **GLI1** | 155 | 0 | 0.00% | 0.00% | 2.35% | 0.00% | 0.00% | 0.00% | 0.00% | 0.00% |

**Supplementary Table 4:** Co-occuring genetic alterations in all mesothelioma cases

| **Gene1** | **Gene2** | **OR** | **pval** | **pval_corrected** |
| --- | --- | --- | --- | --- |
| **CDKN2A** | **CDKN2B** | 2516.42268 | 0 | 0 |
| **CDKN2A** | **MTAP** | 159.8279817 | 6.13694E-81 | 1.18238E-78 |
| **MYC** | **RAD21** | 95.95833333 | 8.10061E-10 | 5.85269E-08 |
| **CDKN2B** | **MTAP** | 78.07467532 | 2.39729E-83 | 6.92815E-81 |
| **EPHA3** | **SETD2** | 9.201342282 | 6.31823E-05 | 0.001922071 |
| **BAP1** | **PBRM1** | 6.789576125 | 2.2463E-21 | 3.24591E-19 |
| **MYC** | **TP53** | 5.397486975 | 3.55122E-05 | 0.001207415 |
| **RB1** | **TP53** | 5.387142857 | 1.90837E-05 | 0.000689399 |
| **BAP1** | **SETD2** | 2.895938567 | 1.05996E-09 | 6.28053E-08 |
| **TERT** | **TP53** | 2.403682512 | 0.000266567 | 0.007336945 |
| **CDKN2A** | **NF2** | 2.099487668 | 2.8979E-11 | 2.79164E-09 |
| **CDKN2B** | **NF2** | 1.998490411 | 1.0866E-09 | 6.28053E-08 |
| **MTAP** | **NF2** | 1.869834711 | 0.000719667 | 0.017331982 |
| **BAP1** | **NF2** | 0.648004765 | 0.000108547 | 0.003136995 |
| **CDKN2B** | **TP53** | 0.599082431 | 0.000554651 | 0.013938612 |
| **BAP1** | **TP53** | 0.4544762 | 3.16706E-08 | 1.66415E-06 |
| **CDKN2A** | **PBRM1** | 0.362866919 | 3.41854E-07 | 1.41137E-05 |
| **CDKN2B** | **PBRM1** | 0.360928363 | 1.74467E-06 | 6.72279E-05 |
| **CDKN2B** | **SETD2** | 0.274950718 | 7.0697E-10 | 5.83755E-08 |
| **BAP1** | **TERT** | 0.26942549 | 8.65509E-08 | 4.16887E-06 |
| **CDKN2A** | **SETD2** | 0.229826353 | 2.12741E-13 | 2.45928E-11 |
| **BAP1** | **RB1** | 0.189956459 | 0.000488219 | 0.012826836 |
| **MTAP** | **SETD2** | 0.103264383 | 3.40255E-07 | 1.41137E-05 |
| **BAP1** | **KRAS** | 0.094703329 | 3.86957E-05 | 0.001242562 |

**Supplementary Table 5:** Co-occuring genetic alterations in pleural mesothelioma cases

| **Gene1** | **Gene2** | **OR** | **pval** | **pval_corrected** |
| --- | --- | --- | --- | --- |
| **CDKN2A** | **CDKN2B** | 3964.235294 | 3.8988E-238 | 1.8617E-235 |
| **CDKN2A** | **MTAP** | 429.2 | 6.90131E-60 | 1.64769E-57 |
| **MYC** | **RAD21** | 74.16666667 | 6.48864E-07 | 3.09833E-05 |
| **CDKN2B** | **MTAP** | 66.70433145 | 7.79594E-58 | 1.24085E-55 |
| **EPHA3** | **SETD2** | 12.87222222 | 5.39332E-05 | 0.001609568 |
| **MYC** | **TP53** | 11.52717391 | 3.0156E-07 | 1.59995E-05 |
| **BAP1** | **PBRM1** | 6.03254149 | 1.66856E-11 | 1.99185E-09 |
| **RB1** | **TP53** | 5.921568627 | 0.000164637 | 0.004367442 |
| **TERT** | **TP53** | 2.966965286 | 6.33646E-05 | 0.001779801 |
| **BAP1** | **SETD2** | 2.627330317 | 2.45094E-06 | 9.00249E-05 |
| **CDKN2A** | **NF2** | 1.843836565 | 2.18129E-06 | 8.67972E-05 |
| **CDKN2B** | **NF2** | 1.736034744 | 1.96174E-05 | 0.000669095 |
| **CDKN2A** | **TP53** | 0.555418719 | 0.000294441 | 0.006695021 |
| **CDKN2B** | **TP53** | 0.537897311 | 0.000185339 | 0.004657854 |
| **BAP1** | **TP53** | 0.416521602 | 1.60993E-07 | 1.0982E-05 |
| **CDKN2B** | **PBRM1** | 0.372918854 | 0.000263002 | 0.006279165 |
| **CDKN2A** | **PBRM1** | 0.335267569 | 2.45018E-05 | 0.000779973 |
| **CDKN2B** | **SETD2** | 0.30941704 | 2.36362E-07 | 1.41079E-05 |
| **CDKN2A** | **SETD2** | 0.264731463 | 9.11696E-10 | 8.7067E-08 |
| **BAP1** | **TERT** | 0.17520362 | 4.328E-09 | 3.44437E-07 |
| **BAP1** | **KRAS** | 0.132264529 | 0.001096437 | 0.023797673 |
| **MTAP** | **SETD2** | 0.078674948 | 1.02538E-06 | 4.4511E-05 |

**Supplementary Table 6**: Co-occuring genetic alterations in peritoneal mesothelioma cases

| **Gene1** | **Gene2** | **OR** | **pval** | **pval_corrected** |
| --- | --- | --- | --- | --- |
| **CDKN2A** | **CDKN2B** | 742.3333333 | 4.68243E-51 | 8.75614E-49 |
| **CDKN2B** | **MTAP** | 109.6923077 | 1.16067E-16 | 1.08523E-14 |
| **CDKN2A** | **MTAP** | 67.275 | 4.32507E-14 | 2.69596E-12 |
| **BAP1** | **PBRM1** | 12.43055556 | 3.43704E-12 | 1.60682E-10 |
| **MTAP** | **NF2** | 4.954545455 | 0.000736866 | 0.012526719 |
| **BAP1** | **SETD2** | 3.856025039 | 0.000403637 | 0.007548012 |
| **CDKN2A** | **NF2** | 3.185349612 | 1.52888E-05 | 0.00040843 |
| **CDKN2B** | **NF2** | 2.887635756 | 0.000222648 | 0.005204402 |
| **BAP1** | **NF2** | 0.332533139 | 1.35311E-05 | 0.00040843 |
| **CDKN2A** | **SETD2** | 0 | 1.22125E-05 | 0.00040843 |
| **SETD2** | **CDKN2A** | 0 | 1.22125E-05 | 0.00040843 |

**Supplementary Table 7**: Mutually exclusive genetic alterations in all mesothelioma cases

| **Gene1** | **Gene2** | **OR** | **pval** | **pval_corrected** |
| --- | --- | --- | --- | --- |
| **CDKN2A** | **SETD2** | 0.25629291 | 3.71009E-15 | 9.8495E-12 |
| **BAP1** | **TP53** | 0.39703852 | 7.03155E-12 | 1.2000E-08 |
| **CDKN2B** | **SETD2** | 0.28761081 | 9.26102E-12 | 1.4752E-08 |
| **BAP1** | **TERT** | 0.24665328 | 1.61139E-09 | 1.9473E-06 |
| **ATM** | **BAP1** | 0.20859319 | 1.91197E-06 | 1.4736E-03 |
| **MTAP** | **SETD2** | 0.19724582 | 2.42258E-06 | 1.7278E-03 |
| **CDKN2B** | **PBRM1** | 0.41450382 | 1.16626E-05 | 6.7964E-03 |
| **BAP1** | **KRAS** | 0.11946234 | 1.64585E-05 | 9.1452E-03 |
| **CDKN2A** | **PBRM1** | 0.45775492 | 2.33682E-05 | 1.1880E-02 |
| **BAP1** | **RB1** | 0.17213473 | 2.44349E-05 | 1.2163E-02 |
| **CDKN2B** | **TP53** | 0.57423547 | 0.000106062 | 4.0224E-02 |

**Supplementary Table 8:** Mutually exclusive genetic alterations in pleural mesothelioma cases

| **Gene1** | **Gene2** | **OR** | **pval** | **pval_corrected** |
| --- | --- | --- | --- | --- |
| **CDKN2A** | **SETD2** | 0.279 | 2.4438E-11 | 3.3307E-08 |
| **BAP1** | **TERT** | 0.170 | 1.6370E-10 | 1.8592E-07 |
| **BAP1** | **TP53** | 0.387 | 1.7624E-09 | 1.5013E-06 |
| **CDKN2B** | **SETD2** | 0.314 | 5.2150E-09 | 4.1809E-06 |
| **MTAP** | **SETD2** | 0.160 | 6.2572E-06 | 3.0457E-03 |
| **ATM** | **BAP1** | 0.185 | 2.3820E-05 | 1.0145E-02 |
| **CDKN2B** | **TP53** | 0.531 | 8.6145E-05 | 3.0105E-02 |
| **CDKN2A** | **PBRM1** | 0.405 | 1.8327E-04 | 4.9404E-02 |

**Supplementary Table 9:** Mutually exclusive genetic alterations in peritoneal mesothelioma cases

| **Gene1** | **Gene2** | **OR** | **pval** | **pval_corrected** |
| --- | --- | --- | --- | --- |
| **BAP1** | **NF2** | 0.3042 | 2.3770E-06 | 1.2780E-04 |
| **CDKN2A** | **SETD2** | 0.0000 | 2.1138E-07 | 1.2880E-05 |
| **CDKN2B** | **SETD2** | 0.0775 | 5.2639E-04 | 1.9245E-02 |

**Supplementary Table 10:** Alterations of *KRAS* in the entire cohort

| **Alteration** | **Count** |
| --- | --- |
| G12C | 10 |
| G12V | 4 |
| G12D | 4 |
| Q61H | 2 |
| G13D | 2 |
| A146T | 1 |
| A146S | 1 |
| G12R | 1 |
| G12S | 1 |
| G13C | 1 |
